# Pattern of cytokine and chemokine production by THP-1 derived macrophages in response to live or heat-killed *Mycobacterium bovis* bacillus Calmette-Guérin Moreau strain

**DOI:** 10.1590/0074-02760140420

**Published:** 2015-09

**Authors:** Periela da Silva Sousa-Vasconcelos, Wellington da Silva Seguins, Eduardo de Souza Luz, Rosa Teixeira de Pinho

**Affiliations:** 1 Fundação Oswaldo Cruz, Instituto Oswaldo Cruz, Laboratório de Imunologia Clínica, Rio de Janeiro, RJ, Brasil; 2 Fundação Ataulpho Paiva, Departamento de Pesquisa, Rio de Janeiro, RJ, Brasil

**Keywords:** BCG, intradermic vaccine, oral vaccine, killed BCG, cytokines, chemokines

## Abstract

Tuberculosis has great public health impact with high rates of mortality and the only prophylactic measure for it is the *Mycobacterium bovis*bacillus Calmette-Guérin (BCG) vaccine. The present study evaluated the release of cytokines [interleukin (IL)-1, tumour necrosis factor and IL-6] and chemokines [macrophage inflammatory protein* (*MIP)-1α and MIP-1β] by THP-1 derived macrophages infected with BCG vaccine obtained by growing mycobacteria in Viscondessa de Moraes Institute medium medium (oral) or Sauton medium (intradermic) to compare the effects of live and heat-killed (HK) mycobacteria. Because BCG has been reported to lose viability during the lyophilisation process and during storage, we examined whether exposing BCG to different temperatures also triggers differences in the expression of some important cytokines and chemokines of the immune response. Interestingly, we observed that HK mycobacteria stimulated cytokine and chemokine production in a different pattern from that observed with live mycobacteria.

*Mycobacterium tuberculosis* (Mtb), the causative agent of tuberculosis (TB), infects approximately one-third of the world’s population. In 2013, nine million people fell ill with TB and 1.5 million died from it, including 360,000 people who were human immunodeficiency virus-positive. In 2013, there were an estimated 480,000 new cases of multidrug-resistant TB (WHO 2015).

There are measures to control TB, such as treatment with antibiotics and, especially, vaccination with *Mycobacterium bovis* bacille Calmette-Guérin 1921 (BCG). BCG, the only licensed TB vaccine, is one of the most widely used vaccines and it is both inexpensive and safe (Kashyap et al. 2010). Approximately 100 million new-born children receive BCG annually because vaccination is consistently protective against childhood TB meningitis and miliary TB. In contrast, BCG efficacy against pulmonary TB in children and adults is highly variable, ranging from 0-80% (Dye 2013).

Initially, the BCG vaccine was given orally; Calmette administered the bacillus culture (BCG vaccine) orally soon after birth (Succi 1985). Later, it was observed that the oral administration of BCG failed to produce an allergic response. Also, a serious accident in Lubeck, Germany occurred in 1930. The oral route was used to vaccinate 250 infants: 73 of them died and another 135 were infected. After this occurred, the vaccine was given *via* the intradermic (ID) route. Twenty months after the accident, it was discovered that the deaths and infections were due to contamination of the vaccine batch with the virulent “Kiel strain” in the Lubeck laboratories (Sakula 1983).

It has also been observed that BCG confers some protection against other mycobacterial infections, such as leprosy, and that it has effects on immunotherapy for some cancers, especially bladder cancer (Herr & Morales 2008). Furthermore, the immunomodulatory effect of BCG was observed in rodents with diabetes type 1 by Faustman et al. (2012). BCG stimulates host innate immunity by inducing the production of cytokines, such as tumour necrosis factor (TNF)-α, which help kill insulin autoreactive T-cells. This form of islet cells can be restored to produce insulin.

Numerous data in both human and mice have shown that protection against TB depends crucially on the response of T-helper (Th)1 type CD4^+^ T-cells, which are paramount in the production of cytokines, such as interferon (IFN)-γ and TNF-α, which are necessary for controlling the infection. These cytokines can activate macrophages to more efficiently destroy the phagocytosed Mtb (Flynn & Chan 2001). TNF-α is an important cytokine in the innate immune response that is produced by macrophages. Macrophages are the primary phagocytic cells involved in the control of Mtb infection and pathogenic and nonpathogenic mycobacteria are internalised by these cells. The interaction of macrophages with various mycobacterial strains induces the production of pro-inflammatory cytokines, such as interleukin (IL)-1β, IL-6, IL-12, TNF-α, granulocyte-macrophage colony-stimulating factor (CSF) and granulocyte-CSF and anti-inflammatory cytokines, including IL-10 (Singh & Goyal 2013).

During the immune response, CC-chemokines, such as macrophage inflammatory protein (MIP)-1α, MIP-1β and regulated on activation, normal T-cell expressed and secreted (RANTES), induce activation and proliferation of T-cells (Taub et al. 1996) and macrophages (Fahey et al. 1992, Lima et al. 1997). The ability of CC-chemokines to attract and activate T-cells and monocytes suggests that chemokines have a role in modulating immune responses to Mtb infection (Méndez-Samperio et al. 2003). According to Fahey et al. (1992), MIP-1α induces macrophage production of TNF-α, IL-1β and IL-6, while MIP-1β modulates MIP-1α-induced TNF-α production. Indeed, monocyte chemotactic protein (MCP)-1 and MIP-1α are known to be potent activators of monocytes/macrophages (Mantovani 1999, Murdoch & Finn 2000).

Nevertheless, because the BCG human vaccine contains living cells, of which an optimal number is essential for the efficacy of BCG vaccination, great care has to be taken to ensure that the vaccine is not exposed to elevated temperatures, even for relatively short periods. Freeze-dried vaccines, such as BCG, have to be stored from +2°C to +8°C (WHO 2010). Because BCG has been reported to lose viability during the lyophilisation process (Lind 1967) and viability is related to storage temperature, in the present work, we studied the possible differences in the immune responses triggered by viable or heat-killed (HK) bacilli with respect to cytokine production. The macrophage is a target cell for these mycobacteria and we evaluated the pattern of cytokines produced by THP-1 derived macrophages after incubation with BCG vaccine obtained by growing mycobacteria in Viscondessa de Moraes Institute (IVM) medium (oral vaccine) or Sauton medium (ID vaccine).

We analysed the levels of the pro-inflammatory cytokines IL-1β, TNF-α and IL-6; the anti-inflammatory cytokine IL-10 and chemokines produced by THP-1 derived macrophages after incubation with live or HK BCG.

The *M. bovis* (BCG) Moreau strain was kindly supplied by the Ataulpho de Paiva Foundation (FAP). The BCG vaccine was utilised at two multiplicity of infection (MOI) of bacillus/THP-1 derived macrophages, 1/1 and 5/1, and the mycobacteria were obtained from two different media, Sauton medium at 37ºC, ID vaccine (Leal et al. 2004) and IVM medium, oral vaccine. Cells from the human THP-1 derived monocytes (acute monocytic leukaemia) were cultivated in a culture flask in RPMI medium containing 10% heat-inactivated foetal calf serum (FCS) (Cutilab cat. n. 6874) inactivated at 37ºC in an atmosphere of 5% carbon dioxide (CO_2_). After growth, cells were removed from the bottle, centrifuged at 4ºC and 300 *g *for 10 min, counted in a Neubauer chamber and distributed in 48-well plates (2 x 10^5^ cells per well) with culture RPMI-1640 medium (LGC cat. n. BR30011.05) and 10% inactivated foetal bovine serum. At 48 h, 30 nM phorbol myristate acetate (PMA) (Sigma cat. n. P1585) was added to differentiate the cells into macrophages based on the method of Spano et al. (2013) with slight modification. The differentiated cells were maintained in supplemented medium without PMA for 48 h before infection. For the infection procedure, we used the lyophilised BCG ID vaccine and oral vaccine.

Mycobacteria were killed by heating them for 3 min at 100ºC, followed by a 3 min cooling step at -20ºC. This procedure was repeated three times HK BCG. The death of these mycobacteria was confirmed by representative bacterial suspensions serially diluted in phosphate-buffered saline (Sigma cat. n. P4417) and colony-forming units were counted after plating on Löwenstein-Jensen medium and 28 days of incubation at 37ºC.

THP-1 derived macrophages were infected at MOIs of 1/1 and 5/1 (bacillus/cell) and were incubated for 3 h at 37ºC with 5% CO_2_. The cells were cultured in RPMI-1640 medium supplemented with 10% FCS and kept at 37ºC with 5% CO_2_. Then, they were washed to remove noninternalised bacilli. Supernatants were removed after 24 h and 72 h of culture and centrifuged at 700 *g* for 5 min at 4ºC in an Eppendorf centrifuge. Samples were aliquoted and stored at -70ºC for the subsequent cytokine and chemokine analyses. The production of cytokines and chemokines, such as MIP-1α and MIP-1β, was measured by enzyme-linked immunosorbent assay. This assay was performed on cell culture supernatants with the duo-set kit from R&D Systems (cat n.: IL-1β, DY 201; IL-6, DY 206; IL-10, DY 217B; MIP-1α, DY 270 and MIP-1β, DY 271). Spectrophotometer (Spectra Max 190) readings were taken at 450 nm. The means and standard error (SE) of the means were calculated and presented in the results as the means ± SE of the means. The response differences between THP-1 cell line-derived macrophages treated with the BCG strain and uninfected controls were compared using the nonparametric Mann-Whitney *U* test (EpiInfo6 v.6.04d; Database and Statistics the Public Health Programme). Statistical significance was set at an alpha level of 5% (p < 0.05).

THP-1 derived macrophages exposed to BCG showed increased production of TNF-α, IL-1β, IL-6 and IL-10 (Fig. 1) in comparison to unexposed THP-1 derived macrophages (controls). We found that treatment of macrophages with all four preparations of BCG resulted in high levels of TNF-α, IL-1β and IL-6. We also observed some differences in the cytokine production patterns between macrophages treated with HK bacillus and live BCG. The cells incubated with ID HK BCG showed a significant differences in TNF-α production at 24 h (*p < 0.05) at the MOI ratio of 1/1 and at 72 h (**p < 0.008) at the MOI of 5/1 (BCG/cell) compared with the cells incubated with ID live BCG. The cells incubated with oral HK BCG at the MOI of 5/1 showed significant differences in TNF-α production at 24 h and 72 h (**p < 0.008) of incubation. IL-1β production by cells treated with ID HK BCG was significantly different at 24 h of incubation at the MOI of 5/1 (*p < 0.05) and at 72 h of incubation at the MOI of 1/1 (*p < 0.05) and the MOI of 5/1 (**p < 0.008). IL-1β production by cells treated with oral HK BCG was significantly different at 24 h and 72 h of incubation at the MOI of 5/1 (**p < 0.008). IL-6 production by cells treated with oral HK BCG was significantly different at 24 h of incubation at the MOI of 1/1 (*p < 0.005). IL-6 production by cells treated with oral HK BCG was significantly different at 72 h of incubation at the MOI of 5/1 (**p < 0.008).

**Fig. 1:**
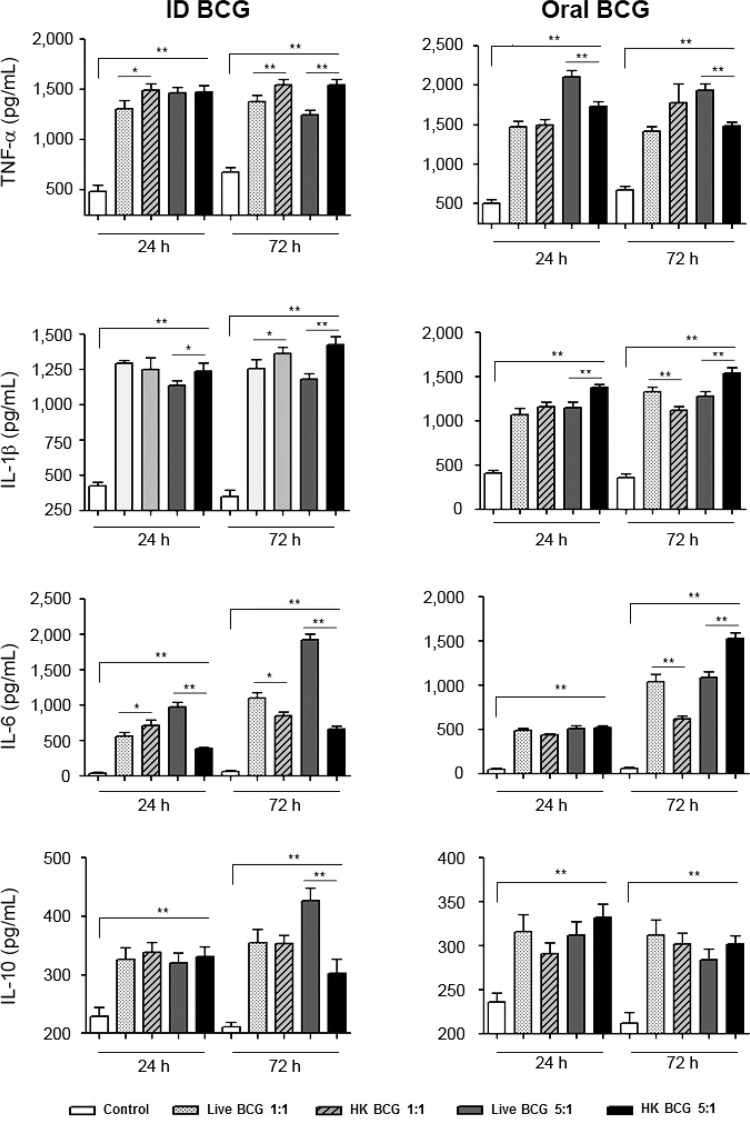
: THP-1 derived macrophages treated with live or heat-killed (HK)*Mycobacterium bovis* bacillus Calmette-Guérin (BCG) produce cytokines. THP-1 derived macrophages were treated with intradermic (ID) or oral BCG Moreau in the multiplicity of infection of 1/1 and 5/1 live or HK mycobacteria. Supernatants from 24 h and 72 h after treatment were removed and the detection of cytokines tumour necrosis factor (TNF)-α, interleukin (IL)-1β, IL-6 and IL-10 were analysed by enzyme-linked immunosorbent assay. Data are expressed as the mean and standard deviation (±) of three independent experiments. Asterisks mean statistical significance (*: p < 0.05; **: p < 0.008, Mann-Whitney*U* test).

There was also a higher production of the cytokine TNF-α in macrophages infected with live oral BCG at the MOI of 5/1 (BCG/cell) compared to the other preparations. Our results are in agreement with those of Suzuki et al. (1993), which showed that BCG is a good inducer of TNF-α, IL-1 and IL-6 production by human and animal macrophages, respectively. Local TNF-α production has been demonstrated to be crucial to granuloma formation (Kindler et al. 1989), which is the host defence mechanism against mycobacterial infection in BCG infected mice

TNF-α is also capable of stimulating the synthesis of IL-10 (Wanidworanun & Strober 1993), a regulatory cytokine that helps control inflammation. We observed an increase in IL-10 production in treated macrophages in comparison to their untreated counterparts (controls), especially at 72 h post-infection in the group of THP-1 derived macrophages infected with ID live BCG at the MOI of 5/1 (BCG/cell). These results partially agree with those published by Moreira et al. (2012), who observed that supernatants of peripheral blood mononuclear cells (PBMCs) exposed to HK or live BCG had similar levels of TNF-α and IL-10.

IL-6 is involved in antibody production and elicits the release of acute-phase proteins (Hirano et al. 1990). We observed increased production of the pro-inflammatory cytokine IL-6 in THP-1 derived macrophages incubated with HK or live BCG. Our results agree with those obtained by Defilippi et al. (1987), who reported that macrophages incubated with HK BCG were just as effective as those infected with live BCG in regards to the production of IL-6. This might be due to the presence of bacillus wall components and bacillus-derived soluble extracts, which act as good inducers of IL-6 (Defilippi et al. 1987). We also speculate that the production of IL-6 and other cytokines by dead bacilli occurs due to the adjuvant effect of mycobacteria on the production of antibodies, as described by Freund (1956). These results are consistent with those presented byHaynes et al. (2004), who showed that the introduction of an adjuvant, such as complete Freund’s adjuvant, which contains killed mycobacteria, induces markedly enhanced host production of lymphotoxin beta, TNF-α, IL-1 and IL-6, independent of age. We observed increased production of IL-6 in THP-1 derived macrophages infected with live ID BCG at the MOI of 5/1 at 72 h (Fig. 1). However, we also observed increased levels of IL-6 in THP-1 derived macrophages incubated with ID HK BCG at the MOI of 5/1 at 72 h. These results may be due to the presence of different components in the media.

Cytokines, such as IL-1, IL-6 and TNF-α, help in the expansion and survival of memory T-cells (Pape et al. 1997, Haynes et al. 2004). Cytokines produced after BCG vaccination may play a role in immune protection because IFN-γ and TNF-α help induce dendritic cell maturation resulting in more effective activation of mycobacteria-specific T-cells (Stenger 2005).

We detected increased production of MIP-1α and MIP-1β in THP-1 derived macrophages infected with live or dead BCG (Fig. 2). The cells incubated with ID and oral HK BCG at the MOI of 1/1 (BCG/cell) showed a significant difference in MIP-1α production after 24 h (*p < 0.008) in comparison with live those incubated with BCG. At 24 h post-treatment, the level of MIP-1α in the supernatants of THP-1 derived macrophages treated with ID live BCG at an MOI of 1/1 was 467 pg/mL vs. 785 pg/mL (*p < 0.008) in THP-1 derived macrophages treated with ID HK BCG (Fig. 2). The cells incubated with oral HK BCG showed significant differences in MIP-1β production at 24 h (*p < 0.05) at the MOI of 1/1 and at 72 h (**p < 0.008) at the MOIs of 1/1 and 5/1 (BCG/cell).

**Fig. 2:**
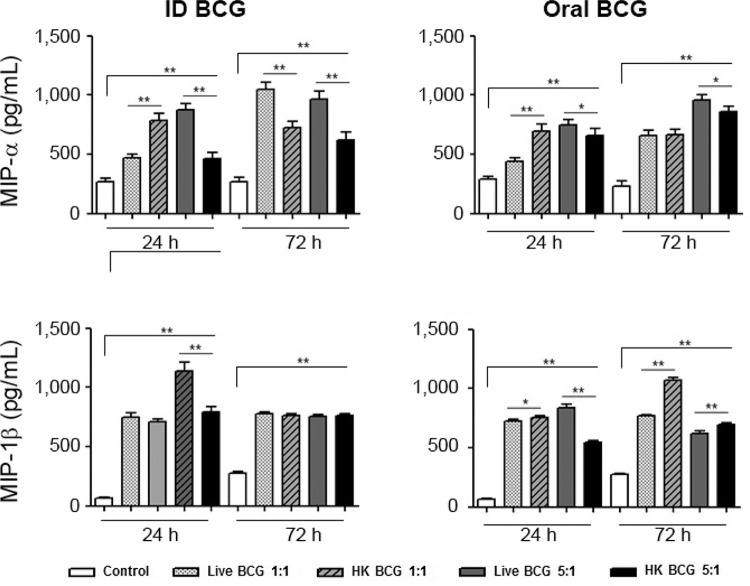
: THP-1 derived macrophages treated with live or heat-killed (HK)*Mycobacterium bovis* bacillus Calmette-Guérin (BCG) produce chemokines. THP-1 derived macrophages were treated with intradermic or oral BCG Moreau in multiplicity of infection of 1/1 and 5/1 live or HK mycobacteria. Supernatants from 24 h and 72 h after treatment were removed and the detection of chemokines macrophage inflammatory protein (MIP)-1α and MIP-1β were analysed by enzyme-linked immunosorbent assay. Data are expressed as the mean and standard deviation (±) of three independent experiments. Asterisks mean statistical significance (*: p < 0.05; **: p < 0.008, Mann-Whitney *U* test).

The secretion of CC-chemokines by human THP-1 derived macrophages infected with BCG may be an important mechanism in BCG-stimulation of the innate immune system. These results are in agreement with those of others studies that reported CC-chemokine secretion by human monocytes in response to BCG infection. Méndez-Samperio et al. (2003) found that biologically significant quantities of RANTES, MIP-1α and MIP-1β were induced by* M. bovis *BCG infection in human monocytes. It is possible that *M. bovis*-induced production of CC-chemokines could activate and regulate several macrophage responses to Mtb. Saukkonen et al. (2002) demonstrated that MIP-1β and RANTES inhibited intracellular growth of Mtb. Also, Mohammed et al. (1999) demonstrated that Th1 and Th2 cytokines regulate CC-chemokine expression in mouse pleural mesothelial cells (PMCs) and that in vitro stimulation of PMCs with HK BCG results in MIP-1α and MCP-1 mRNA expression.

In this study, we found that HK mycobacteria induced a cytokine response that showed minor differences to that induced by infection with viable bacilli. This could be due to the activation of macrophage phagocytosis, which occurs in the presence of mycobacteria and also because mycobacteria antigens, which are necessary for activation of the immune response, were maintained after the mycobacteria were HK. Our data also support some previous experimental results in regards to the use of BCG as an adjuvant in immunotherapy for superficial bladder urothelial carcinoma *“in situ”*and as a future therapy for diabetes mellitus type 1 (Herr & Morales 2008, Faustman et al. 2012). Immunogenicity, a high proportion of live bacilli, multiplication and persistence of BCG in host tissues are important requirements of BCG vaccine efficacy. In this regard, our work showed that incubation of THP-1 derived macrophages with live or HK mycobacteria of the BCG Moreau strain both resulted in the production of pro-inflammatory cytokines, such as IL-1β, TNF-α and IL-6 and the chemokines MIP-1α and MIP-1β (Fig. 2). When we compared the production of cytokines between THP-1 derived macrophages incubated with live or dead bacteria, we found that the ones incubated with live bacteria produced significantly more cytokines than their HK counterparts. Because cell integrity and active metabolism trigger cytokine production (Secanella-Fandos et al. 2014), the differences observed here may be due to differences in antigens between the live and HK BCG.

Secanella-Fandos et al. (2014) compared irradiated and HK BCG with live BCG and observed that some of the molecules responsible for inducing cytokine production are clearly thermosensitive. Their results suggest that killed but metabolically active BCG (irradiated) could be a safer immunotherapy alternative to live bacillus treatment. The minor production of cytokines with HK BCG observed here may be due to the loss of some thermosensitive antigens. It is not yet clear if bacterial antigens are lost during improper storage of BCG Moreau vaccine, but our results suggest that in the presence of dead mycobacteria, the vaccine may still induce the production of cytokines and chemokines involved in immune responses.

Some limitations of the present study include the use of THP-1 derived macrophages and in vitro* “*immune responses”, which may change according to cell type (Daigneault et al. 2010). Our results suggest that the BCG Moreau vaccine has good immunogenicity and that dead bacteria can also participate in this response.

The BCG vaccine has great significance, not only as a control measure for TB, which is still considered a public health problem, but also as an immunomodulator in other pathologies. Killed BCG may be a promising alternative to live BCG because negative side effects due to mycobacterial infection can be ruled out. However, several immunological mechanisms triggered by live and killed microorganisms in human hosts require further elucidation.

## References

[B1] Daigneault M, Preston JA, Marriott HM, Whyte MK, Dockrell DH (2010). The identification of markers of macrophage
differentiation in PMA-stimulated THP-1 cells and monocyte-derived
macrophages. PLoS ONE.

[B2] Defilippi P, Poupart P, Tavernier J, Fiers W, Content J (1987). Induction and regulation of mRNA encoding 26-kDa protein
in human cell lines treated with recombinant human tumor necrosis
factor. Proc Natl Acad Sci USA.

[B3] Dye C (2013). Making wider use of the world’s most widely used
vaccine: bacille Calmette-Guerin revaccination reconsidered. J R Soc Interface.

[B4] Fahey T, Tracey K, Tekamp-Olson P, Cousens L, Jones W, Shires G, Cerami A, Sherry B (1992). Macrophage inflammatory protein-1 modulates macrophage
function. J Immunol.

[B5] Faustman DL, Wang L, Okubo Y, Burger D, Ban L, Man G, Zheng H, Schoenfeld D, Pompei R, Avruch J, Nathan DM (2012). Proof-of-concept, randomized, controlled clinical trial
of bacillus-Calmette-Guerin for treatment of long-term type 1
diabetes. PLoS ONE.

[B6] Flynn JL, Chan J (2001). Immunology of tuberculosis. Annu Rev Immunol.

[B7] Freund J (1956). The mode of action of immunologic
adjuvants. Bibl Tuberc.

[B8] Haynes L, Eaton SM, Burns EM, Rincon M, Swain SL (2004). Inflammatory cytokines overcome age-related defects in
CD4 T-cell responses in vivo. J Immunol.

[B9] Herr HW, Morales A (2008). History of bacillus Calmette-Guerin and bladder cancer:
an immunotherapy success story. J Urol.

[B10] Hirano T, Akira S, Taja T, Kishimoto T (1990). Biological and clinical aspects of interleukin
6. Immunol Today.

[B11] Kashyap RS, Husain AA, Morey SH, Panchbhai MS, Deshpande PS, Purohit HJ, Taori GM, Daginawala HF (2010). Assessment of immune response to repeat stimulation with
BCG vaccine using in vitro PBMC model. J Immune Based Ther Vaccines.

[B12] Kindler V, Sappino AP, Grau GE, Piguet PF, Vassalli P (1989). The inducing role of tumor necrosis factor in the
development of bactericidal granulomas during BCG infection. Cell.

[B13] Leal MBB, Baruque-Ramos J, Hiss H, Paz MF, Sakai MC, Vassoler UM, Arauz LJ, Raw I (2004). Influence of initial L-asparagine and glycerol
concentrations on the batch growth kinetics of Mycobacterium bovis
BCG. Braz J Microbiol.

[B14] Lima M, Zhang Y, Villalta F (1997). β-chemokines that inhibit HIV-1 infection of human
macrophages stimulate uptake and promote destruction of Trypanosoma cruzi by human
macrophages. Cell Mol Biol.

[B15] Lind A (1967). Stability of dried BCG vaccine stored at -70º, -25º and
+40ºC. Scand J Respir Dis.

[B16] Mantovani A (1999). The chemokine system: redundancy for robust
outputs. Immunol Today.

[B17] Méndez-Samperio P, Vázquez A, Ayala H (2003). Infection of human monocytes with Mycobacterium bovis
BCG induces production of CC-chemokines. J Infect.

[B18] Mohammed KA, Nasreen N, Ward MJ, Antony VB (1999). Helper T-cell type 1 and 2 cytokines regulate
CC-chemokine expression in mouse pleural mesothelial cells. Am J Respir Crit Care Med.

[B19] Moreira J, Aragão WC, Barillas SG, Barbosa SM, Pedroza LA, Condino A (2012). Human leucocytes response to viable extended
freeze-drying or heat-killed Mycobacterium bovis bacillus
Calmette-Guérin. Scand J Immunol.

[B20] Murdoch C, Finn A (2000). Chemokine receptors and their role in inflammation and
infectious diseases. Blood.

[B21] Pape KA, Khoruts A, Mondino A, Jenkins MK (1997). Inflammatory cytokines enhance the in vivo clonal
expansion and differentiation of antigen-activated
CD4^+^T-cells. J Immunol.

[B22] Sakula A (1983). BCG: Who were Calmette and Guérin?. Thorax.

[B23] Saukkonen JJ, Bazydlo B, Thomas M, Strieter RM, Keane J, Kornfeld H (2002). Beta-chemokines are induced by Mycobacterium
tuberculosis and inhibit its growth. Infect Immun.

[B24] Secanella-Fandos S, Noguera-Ortega E, Olivares F, Luquin M, Julián E (2014). Killed but metabolically active Mycobacterium bovis
bacillus Calmette-Guérin retains the antitumor ability of live bacillus
Calmette-Guérin. J Urol.

[B25] Singh PP, Goyal A (2013). Interleukin-6: a potent biomarker of mycobacterial
infection. Springerplus.

[B26] Spano A, Barni S, Sciola L (2013). PMA withdrawal in PMA-treated monocytic THP-1 cells and
subsequent retinoic acid stimulation, modulate induction of apoptosis and
appearance of dendritic cells. Cell Prolif.

[B27] Stenger S (2005). Immunological control of tuberculosis: role of tumour
necrosis factor and more. Ann Rheum Dis.

[B28] Succi RCM, Farhat CK (1985). BCG. Fundamentos e prática das imunizações em clínica médica e
pediatria.

[B29] Suzuki K, Fukutomi Y, Matsuoka M, Toni K, Hayashi H, Takii T, Oomoto Y, Onozaki K (1993). Differential production of interleukin 1 (IL-1), IL-6,
tumor necrosis factor and IL-1 receptor antagonist by human monocytes stimulated
with Mycobacterium leprae and M. bovis BCG. Int J Lepr.

[B30] Taub D, Turcovski-Corrales S, Key M, Longo D, Murphy W (1996). Chemokines and T lymphocyte activation: β-chemokines
costimulate human T lymphocyte activation in vitro. J Immunol.

[B31] Wanidworanun C, Strober W (1993). Predominant role of tumor necrosis factor-alpha in human
monocyte IL-10 synthesis. J Immunol.

[B32] WHO (2010). Vaccine handling: proper handling and reconstitution of
vaccines avoids programme errors.

[B33] WHO (2015). The end TB strategy.

